# Personalized Treatment of Recurrent, Metastatic Head and Neck Cancer Guided by Patient-Derived Xenograft Models

**DOI:** 10.7759/cureus.53645

**Published:** 2024-02-05

**Authors:** Morgan D Black, John Yoo, Kevin Fung, Danielle MacNeil, David A Palma, Joseph S Mymryk, Sara Kuruvilla, John W Barrett, Eric Winquist, Anthony C Nichols

**Affiliations:** 1 Medical Oncology, London Health Sciences Centre, London, CAN; 2 Otolaryngology - Head and Neck Surgery, Western University, London, CAN; 3 Otolaryngology - Head and Neck Surgery, London Health Sciences Centre, London, CAN; 4 Radiation Oncology, London Health Sciences Centre, London, CAN; 5 Microbiology, Western University, London, CAN

**Keywords:** personalized medicine, patient-derived xenograft models, chemotherapy, recurrent/metastatic head and neck squamous cell carcinoma, head and neck cancer

## Abstract

Recurrent or metastatic head and neck squamous cell carcinoma (RMHNSCC) is associated with a poor prognosis and short survival duration. There is an urgent need to identify personalized predictors of drug response to guide the selection of the most effective therapy for each individual recurrence. We tested the feasibility of patient-derived xenografts (PDX) for guiding their RMHNSCC salvage treatment. Fresh tumor samples from eligible, consented patients were implanted into mice. Established tumors were expanded in mouse PDX cohorts to identify responses to candidate salvage drug treatments in parallel testing. Patients alive and suitable for chemotherapy were treated based on responses determined by PDX testing. Nine patient tumors were successfully engrafted in mice with an average time of 89.2±41.7 days. Four patients' PDX models underwent parallel drug testing. Two patients received PDX-guided therapy. In one of these patients, single agents of cetuximab and paclitaxel demonstrated the best responses in the PDX model, and this patient exhibited sequential partial responses to each drug, including a 17-month clinical response to cetuximab. The main limitation of PDX testing for RMHNSCC was the time delay in obtaining testing results. Despite this, parallel PDX testing may be feasible for a subset of patients and appears to correlate with clinical benefit.

## Introduction

Head and neck cancer squamous cell carcinoma (HNSCC) is the sixth most common cancer worldwide, reporting more than 500,000 new cases and 300,000 deaths annually [1]. Despite multi-modality treatment with surgery, radiation, and/or chemotherapy, the recurrence and metastatic rates for HNSCC remain high, and patients suffering relapse remain at extremely high risk of dying from the disease [2, 3]. Often, palliative chemotherapy is the only option; however, response rates to conventional chemotherapies remain poor, with combination chemotherapy response rates at approximately ~25% [4] and approximately 35% when chemotherapy is combined with cetuximab [5].

RMHNSCC is associated with high morbidity and a poor prognosis, with a median overall survival of approximately seven months [6]. As opposed to other solid tumor cancers, the list of FDA- and Health Canada-approved chemotherapies for head and neck cancer was very limited before the advent of immunotherapy, including only cisplatin, carboplatin, Taxotere, 5-fluorouracil, methotrexate and cetuximab (monoclonal antibody against the epidermal growth factor receptor) [7]. A meta-analysis performed by Winquist and colleagues examined randomized clinical trials involving these agents alone or in combination and found that these agent(s) resulted in minimal or no impact on patient survival or quality of life (QOL) [4]. Although significant responses are seen in a small subset of patients that can prolong survival and decrease symptoms, this is balanced by the toxicity experienced in the remainder of patients that results in poorer QOL and possibly a faster demise. As such, there is an urgent need for a mechanism to identify which patients respond to each treatment that would allow the delivery of drugs only to those that are likely to respond.

Drug responses in patient-derived xenografts (PDX) models, particularly Champions Oncology TumorGraft® models (Champions Oncology Inc., Hackensack, New Jersey), have been highly correlated with tumor responses in patients [8-10]. These models have been used in an experimental setting to guide care and have produced long-term responders in a subset of patients, sometimes lasting years [10]. However, a randomized trial comparing response rates and survival in TumorGraft®-directed therapy vs. standard of care has never been carried out and would be required to definitively prove that this approach provides clinical benefit above empiric chemotherapy delivery. If a significant survival benefit can be demonstrated, TumorGraft®-directed therapy may establish a new standard of care for patients with RMHNSCC. In this study, we sought to test the feasibility of personalized patient-derived tumor xenograft models for guiding systemic treatment in RMHNSCC patients. 

## Materials and methods

Research ethics board approval was obtained from Western University in London, Ontario, Canada (REB#: 106515). Fresh biopsies (minimum of 4 x 18-gauge biopsies 10 mm in depth) and/or surgical specimens (≥0.5 cm^3^ non-minced) were collected from patients and immediately placed into media and shipped on ice. In partnership with Champions Oncology (Hackensack, New Jersey), these samples were then sent to their Mississauga, Ontario, Canada location, where they were implanted into immunocompromised NOG mice (Taconic Biosciences Inc, New York City, New York) on the same day as tissue retrieval for construction of PDX models using the Champions Oncology animal use protocol (CO#010) as previously described [8]. 

Chemotherapeutic drugs

Chemotherapy agents used in this study included 5-fluorouracil, capecitabine, carboplatin, cetuximab, cisplatin, doxorubicin, GDC0032, gemcitabine, methotrexate, and paclitaxel and were supplied by Champions Oncology Inc. and formulated according to the manufacturer's specifications. The dose, schedule, and route of administration for each drug were established based on prior Champions Oncology studies [11] and are listed in the Appendix Table 5.

Patient-derived xenografts

The Champions Personalized TumorGraft® tests were conducted on TumorGraft® models established from fresh RMHNSCC patient tumor samples collected from either a fresh biopsy or surgical sample. A range of 5-15 mice were implanted from each patient sample based on the amount of fresh tumor samples received. 

The percentage of mice that were engrafted from each sample was recorded. For samples that engrafted, the TumorGraft® tumors were excised, cut into 1mm^3^ pieces, and passaged into second-generation models for drug testing. A maximum of four drugs were selected by the treating oncologist (EWW, SR, or MSK) to be tested. Drug treatment was initiated when patient TumorGraft® models reached approximately 200mm3. The designated endpoints for each model were an estimated tumor volume (TV) of approximately 1000mm^3^ or 21 days following treatment initiation. Beginning day zero, tumor dimensions were measured twice weekly using digital calipers, and growth data, including individual and mean estimated tumor volumes (mean TV ± SEM), were recorded for each treatment group. Tumor volume was calculated using the formula: TV=width² x length x π/6.

At test completion, percent tumor growth inhibition (%TGI) values were calculated and reported for each treatment group (T) versus the control group (C) using initial tumor measurements (i) and final tumor measurements (f) by the formula: %TGI=(1-(Tf-Ti)/(Cf-Ci)) x 100. Percent tumor regression (%TR) values were calculated for all animals using the formula: %TR= (1-Tf/Ti) x 100. A mean %TR value was calculated for the entire treatment group. At test completion, treatment groups showing an average TR <-120% of the day zero measurement were considered to have progressive disease (PD). Treatment groups showing an average TR between -120% and 30% of the day zero measurement were considered to have stable disease (SD). Treatment groups showing an average TR of ≥30% of the day zero measurement were considered to have a partial response (PR). Individual mice lacking palpable tumors for at least two consecutive measurements at the completion of dosing were classified as complete responders, having demonstrated a complete response (CR). Drugs or combinations showing overall SD with a high TGI, PR, or CR were considered to have potential benefits. Drugs or combinations showing overall PD were considered to show unlikely benefit. Drugs or combinations showing SD with a low tumor growth inhibition (TGI) value or inconclusive data were considered indeterminate.

## Results

Characteristics of RMHNSCC patients

A total of 13 patients consented to the study, with 10 patients successfully having tumor samples collected (Table 1). The majority of patients were male (80%, n=8) with tumors primarily in the oropharynx (50%, n=5) and oral cavity (20%, n=2). Eighty percent of tumors implanted were squamous cell carcinomas, with other histologies including adenoid cystic carcinoma (10%, n=1) and a high-grade sarcomatoid carcinoma (10%, n=1). As per the American Joint Committee on Cancer (AJCC) eighth edition staging, all patients had advanced disease (clinical stage III or IV) at the time of tumor tissue collection. Only one of the 10 patients was treatment-naïve, while all other patients had received prior surgery, chemotherapy, and/or radiation therapy (Table 1). Common sites of disease relapse for these patients were the neck (38%) and lungs (14%), with a smaller number of patients also recurring in the bone, lymph nodes, salivary gland, liver, dermis, and axilla or supraclavicular area.

**Table 1:** Baseline patient demographics of patients whose tumor tissue was collected to be engrafted AJCC - American Joint Committee on Cancer

Characteristic	N (%)
Age at testing (years)	60 ± 7.1 (SD)
Sex	
Male	8 (80)
Female	2 (20)
Primary tumor site	
Base of tongue	3 (30)
Tonsil	2 (20)
Oral cavity	2 (20)
Salivary gland	1 (10)
Maxilla	1 (10)
Neck	1 (10)
Histology	
Squamous cell carcinoma (SCC)	8 (80)
Adenoid cystic carcinoma	1 (10)
Sarcomatoid carcinoma	1 (10)
T Stage	
T1	1 (10)
T2	2 (20)
T3	1 (10)
T4	4 (40)
T4a	1 (10)
Unknown	1 (10)
N Stage	
N0	2 (20)
N1	1 (10)
N2	1 (10)
N2b	2 (20)
N2c	2 (20)
N3	1 (10)
Unknown	1 (10)
M Stage	
M0	6 (60)
M1	2 (20)
Unknown	2 (20)
Clinical Stage (AJCC 8^th^ edition)	
III	3 (30)
IVA	2 (20)
IVA/B	2 (20)
IV	1 (10)
IVC	1 (10)
UNK	1 (10)
Prior treatment(s)	
Surgery?	
No	5 (50)
Yes	5 (50)
Radiation?	
No	1 (10)
Yes	9 (90)
Chemotherapy?	
No	2 (20)
Yes	8 (80)
Site(s) of relapse	
Neck	5 (38)
Bone	1 (8)
Lung	2 (14)
Lymph node	1 (8)
Salivary gland	1 (8)
Liver	1 (8)
Dermis	1 (8)
Axilla/supraclavicular	1 (8)

Patient-derived tumor xenograft models

**Figure 1:**
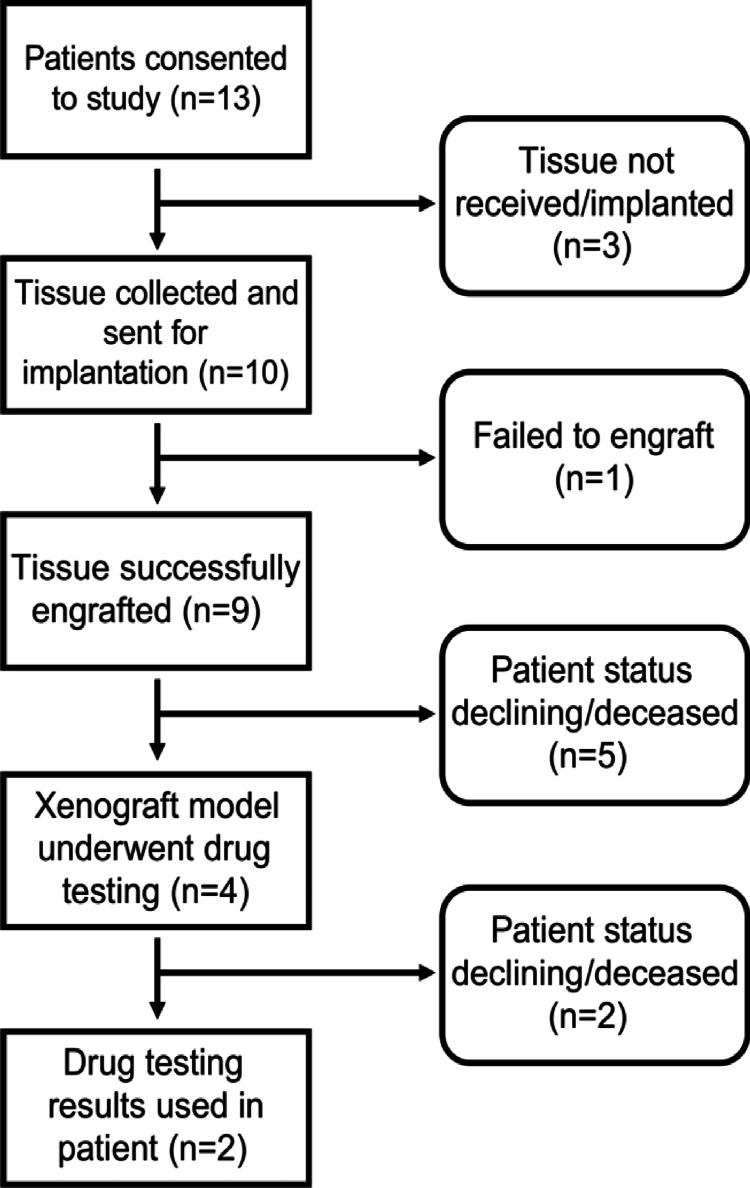
Flowchart of study workflow; the number of individual unique patient-derived samples present at each stage is indicated in brackets

A flowchart of the study recruitment process is shown in Figure 1. Fresh tumor tissue was collected from 10 patients and implanted into immunocompromised mice. The average number of implants performed for each sample was 10.4 ± 3.8 mice. Samples were implanted on the same date as the tumor harvest date, except for LON-012, which was implanted the following date after harvest, due to a shipping delay.

Of the 10 patients with viable tumor tissue, nine samples (90%, n=9) successfully engrafted into mice. The average time to engraftment was 89.2 ± 41.7 days (Table 2). The successfully engrafted samples consisted of squamous cell carcinoma (89%) and sarcomatoid carcinoma (11%). The majority of samples were biopsy samples (90%, n=9) from metastatic sites, including the neck and liver, as well as primary sites, such as the base of the tongue and mandible (Table 2).

**Table 2:** Histological classification and procedure history information of implanted tumor tissue samples SCC - squamous cell carcinoma; ACC - adenoid cystic carcinoma *This patient was discontinued as their tumor tissue did not graft

Patient ID	Histology	Specimen site	Procedure type	Number of mice implanted	Days to engraftment	Drug testing completed?
LON-001	SCC	Base of tongue	Surgery	9	127	No
LON-002	SCC	Dermal neck	Biopsy	12	71	Yes
LON-003*	ACC	Base of tongue	Surgery	15	N/A	N/A
LON-004	SCC	Mandible	Biopsy	6	97	No
LON-006	SCC	Neck	Biopsy	9	78	No
LON-007	SCC	Liver	Biopsy	5	78	No
LON-008	SCC	Neck	Biopsy	11	71	Yes
LON-009	SCC	Neck	Open biopsy	15	52	Yes
LON-011	Sarcomatoid carcinoma	Neck	Open biopsy	15	181	Yes
LON-012	SCC	Neck	Biopsy	7	48	No

Patient status following xenograft engraftment

Of the nine successfully established xenograft models, four PDX models underwent drug testing, as patients were alive and deemed well enough to receive chemotherapy at the point that the second generation TumorGrafts® were large enough for testing (Table 3). Three of these four patients were alive at the time their drug testing was completed and results were available (Table 3). Unfortunately, the models derived from patient LON-009 and LON-011's tumor tissue did not respond to any of the chemotherapeutic agents tested (Appendix Table 5).

**Table 3:** Status of patients with successfully engrafted tumor tissue at engraftment completion and drug testing completion PDX - patient-derived xenografts *Patients that were alive at the completion of PDX drug testing

Patient ID	Engraftment completion date	Patient status at engraftment completion	Drug testing completion date (if applicable)	Patient status at completion of drug testing
LON-001	2016/03/30	Likely deceased	N/A	N/A
LON-002*	2016/03/18	Alive	2016/06/14	Alive
LON-004	2016/07/06	Alive (declining)	N/A	N/A
LON-006	2016/06/30	Alive (declining)	N/A	N/A
LON-007	2016/08/11	Deceased	N/A	N/A
LON-008*	2016/08/11	Alive	2016/10/13	Alive
LON-009	2017/01/19	Alive	2017/06/30	Deceased
LON-011*	2017/10/03	Alive	2018/10/23	Alive
LON-012	2017/11/01	Alive	N/A	N/A

Patients LON-002 and LON-008 were both alive at the completion of their personalized PDX model drug testing and were deemed eligible to receive chemotherapy by their treating oncologist. Drug testing of LON-002's xenograft demonstrated that treatment with paclitaxel (Appendix Table 6) displayed potential benefit as it resulted in one animal showing a PR and two animals showing SD (Table 4). Testing of the xenografts generated from LON-008 revealed that the single agents cetuximab and paclitaxel, as well as the combination of paclitaxel/cetuximab, demonstrated potential benefit, as they were able to inhibit tumor growth and produce an overall response of SD (Appendix Table 6).

**Table 4:** Results of chemotherapeutic drug testing of LON-008 patient-derived xenografts. TGI-Tumor Growth Inhibition; TR-Tumor Response

Agent	n	%TGI	%TR	Overall Response
Control	3	--	-196	PD
Methotrexate	3	14	-152	PD
Cetuximab	3	107	13	SD
Paclitaxel/ Cetuximab	3	105	11	SD
Gemcitabine	3	65	-81	PD
Paclitaxel	3	93	-17	SD

Of the four patients who had parallel drug testing completed on their patient-derived xenografts, the average time from engraftment completion to drug testing completion was 83.8 ± 59.9 days. Taken together, the total time from tumor implantation into mice to completion of drug testing in patient-derived xenografts was 177.5 ± 37 days.

LON-002 was on trial with an immunotherapy agent when drug testing was completed. PDX drug testing demonstrated that paclitaxel may be beneficial, and LON-002 was able to receive one cycle of the recommended single-agent paclitaxel. Unfortunately, the patient was unable to continue treatment due to rapid deterioration in their functional status. LON-002 succumbed to their disease before paclitaxel treatment could be resumed.

LON-008: a case of real-time personalized patient-derived xenograft models guiding systemic treatment in RMHNSCC

Patient LON-008 presented to the Head and Neck Multi-disciplinary team in January 2014, with a T2N1M0 squamous cell carcinoma of the left tongue. The patient underwent a hemiglossectomy and neck dissection in March 2014, followed by adjuvant post-operational radiation therapy from March to May 2014. Unfortunately, the tumor recurred in January 2015 in the left parapharyngeal space, as well as in the upper neck abutting the carotid, and this was deemed unresectable.

In the metastatic setting, LON-008 received first-line combination cisplatin and 5-fluorouracil chemotherapy from March to June 2015 for a total of five cycles. This regimen was discontinued due to an episode of febrile neutropenia, fatigue, and nausea. Second-line systemic therapy of weekly paclitaxel was administered from July to August 2016 for a total of seven cycles. This, however, had to be discontinued due to fatigue and malaise. Fortunately, CT imaging in October 2016 revealed a PR to therapy.

**Figure 2:**
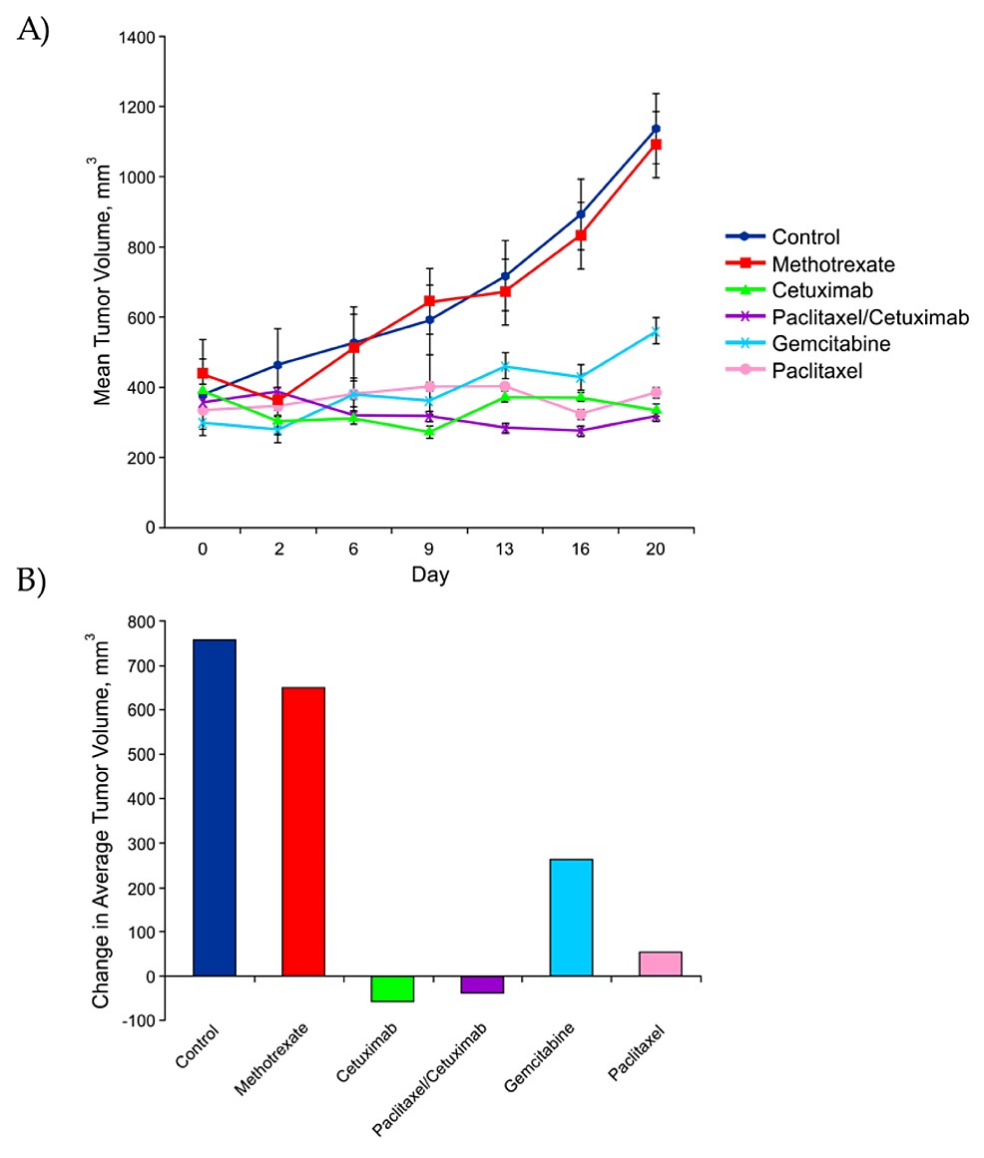
LON-008 patient-derived xenograft tumor growth (A) Average growth of LON-008 xenograft model treatment groups at test completion; (B) the relative changes in LON-008 derived xenograft tumor volume at study termination.

In October 2016, the results of LON-008's patient-derived xenograft drug testing were received, which indicated the potential benefit of single agents cetuximab and paclitaxel as well as combination cetuximab/paclitaxel (Table 4, Figure 2). These results were supported by the four-month clinical response observed in LON-008 following treatment with paclitaxel, which was not discontinued based on efficacy but due to toxicity. Based on the PDX drug testing results, it was decided that single-agent cetuximab would be used next in the third-line setting.

LON-008 began third-line weekly cetuximab in October 2016 and continued for 17 months until February 2018, with treatment breaks due to fatigue. Complete responses by CT imaging were mostly observed throughout this period. However, a recurrence was observed on imaging in September 2017, following a four-month treatment break and weekly cetuximab was resumed until February 2018 with a good response. Eventually, the patient progressed and was switched to fourth-line biweekly nivolumab in March 2018, eventually switching to monthly nivolumab in October 2018. LON-008 has experienced continued SD on fourth-line nivolumab until June 2019. This was discontinued in July 2019 due to pneumonitis. However, the patient has remained stable on surveillance as of June 2020.

## Discussion

Targeted therapies are an emerging therapeutic strategy in HNSCC that target a specific genetic abnormality that has been identified, and recent prognostic signatures have been reported, particularly for human papillomavirus-positive disease [12]. Important molecular targets in HNSCC include cell surface receptors estimated glomerular filtration rate (eGFR), HER-2, and vascular endothelial growth factor receptor (VEGFR), as well as proteins in the PI3K/Akt/mTOR, mitogen-activated protein kinase (MAPK), and Janus kinase-signal transducer and activator of transcription (JAK-STAT) pathways [13]. RMHNSCC has increasingly been managed with immunotherapy. Nivolumab received FDA approval in November 2016 and Health Canada approval in May 2017 based on the CheckMate 141 trial results [14]. Similarly, Pembrolizumab received FDA approval in June 2019 based on the KEYNOTE-012 trial. Unfortunately, despite these promising advancements, outcomes remain poor for most RMHNSCC patients, with the possible exception of human papillomavirus-positive patients with local/locoregional recurrence [15], illustrating the need for novel therapeutic options as well as validated biomarkers and reliable models to help guide treatment.

In an effort to address this, and with the knowledge that the median survival in patients with RMHNSCC is approximately seven months, we aimed to evaluate the feasibility of generating personalized patient-derived xenograft models for real-time parallel evaluation of chemotherapeutic agents, with the goal of guiding systemic salvage treatment in RMHNSCC patients. In our study, the average time to engraftment was approximately 2.9 months (n=9), and the average time from engraftment to drug testing completion was 2.8 months (n=4). Therefore, the total time from RMHNSCC tumor tissue implantation into mice to completion of parallel drug testing was approximately 5.8 months (n=4). When compared to the median survival of seven months for RMHNSCC patients, there are challenges with obtaining PDX drug testing results, as some patients may not be alive when testing results are available, and many others may not be healthy enough to receive the recommended systemic treatments. Despite this, our study found that generating PDXs for RMHNSCC is certainly feasible for select patients.

Our engraftment rate of fresh tumor tissue into mice was 90% (n=9). All successfully engrafted samples were squamous cell carcinomas, whereas the one sample that did not take in mice was adenoid cystic carcinoma (ACC) histology. Despite having recurred with their ACC, patient LON-003's disease has remained relatively indolent since their recurrence in 2015. LON-003 received minimal systemic therapy from late 2016 to early 2019 with slow progression of previously identified lesions. This lack of disease progression might represent a less aggressive tumor, which could be a contributory factor to its lack of successful engraftment into mice.

One of the challenges of real-time systemic drug testing of tumor tissue presents is the appropriate selection of chemotherapeutic agent(s) and combination(s) for testing in PDX mouse models. In this study, the selection of systemic agents for simultaneous parallel testing was made by the treating medical oncologist. This selection process was typically made by testing approved and funded agents and taking into consideration all applicable information concerning the patient's condition. Additionally, some drugs were selected based on previous exposure to validate the observed response(s) of previous treatment(s).

It is important to note that this study started prior to immunotherapy being approved and accessible in Canada. With immunotherapy agents such as nivolumab receiving approval and becoming funded for HNSCC in Canada, the importance of testing these agents is evident based on positive results from trials leading to its approval. Unfortunately, the current shortcomings and technical difficulties of testing immunotherapy drugs in mouse models remain a costly and complex scientific challenge. Moving forward, continued advancements in humanized mouse models, which harbor human immune cells that can respond to treatment with immunotherapy agents, will enable more applicable and comprehensive future research in PDX drug testing for RMHNSCC, as well as other cancer types as immunotherapy agents continue to receive approval for various disease sites [16].

Real-time patient-derived xenograft drug testing of patient LON-008 in our cohort displayed the potential benefits of single agents cetuximab and paclitaxel, as well as combination cetuximab/paclitaxel for this particular patient. The personalized PDX drug testng results for this patient recapitulated a previously used and successful chemotherapeutic agent, paclitaxel, aided the oncologists in the selection of their next line of therapy as well. Based on the results of their PDX drug testing, LON-008 began third-line cetuximab and demonstrated a durable response for approximately 17 months, following a positive response to PDX-supported paclitaxel.

There were several limitations to this study. For example, it was challenging to obtain PDX drug testing results before the deterioration of the patient's health made further treatment untenable. As well, although this strategy allows for simultaneous real-time testing of multiple chemotherapeutic agent(s) and combination(s) of agents the results are still limited by the information generated from an immunodeficient murine background. Finally, the number of drugs tested or combinations of chemotherapy evaluated is limited by the number of amplified PDX models that were established.

## Conclusions

Currently, patients with RMHNSCC have limited options in terms of systemic therapy. Cisplatin, carboplatin, 5-fluorouracil, methotrexate, docetaxel, cetuximab, and nivolumab (second line) are approved and funded in Canada for this indication, but response rates and survival benefits remain poor in most individuals. Novel approaches for treating RMHNSCC that recognize the heterogeneity of this population and provide more personalized biomarkers of treatment response are needed to identify which patient groups may benefit from a given systemic regimen. Although this study only yielded one case where simultaneous parallel PDX drug testing was able to successfully validate and guide therapy, it offers insights into the potential utility of real-time PDX models for guiding systemic therapy in RMHNSCC. In the future, as our understanding of HNSCC and response to therapy increases, so too will our ability to guide systemic therapy options in real-time for patients requiring treatment. Moreover, xenograft-directed treatment recommendations can be utilized to improve outcomes in any cancer type, as this strategy promises to identify and avoid unnecessary, costly chemotherapy treatments that will be futile, and perhaps even deleterious, for an individual patient while identifying the most effective regimen to provide personalized care.

## Appendices

**Table 5:** The dose, schedule, and route of administration for chemotherapeutic agents used in this study IP - intraperitoneal; PO - oral administration; IV - intravenous

Drug	Dose (mg/kg/dose)	Schedule	Route of administration
5-fluorouracil	50	q7dx3	IP
Capecitabine	200	qdx21	PO
Carboplatin	25	q7dx3	IP
Cetuximab	20	biwx3	IP
Cisplatin	5	q7dx3	IP
Doxorubicin	3	q7dx3	IV
GDC0032	5	qdx21	PO
Gemcitabine	100	q7dx3	IP
Methotrexate	5	q3dx4	IP
Paclitaxel	20	q7dx3	IV

**Table 6:** Tumor growth and response of drug-tested patient-derived xenografts TGI - tumor growth inhibition; TR - tumor response; PD - progressive disease; SD - stable disease; PR - partial response; CR - complete response *Chemotherapeutic agents that demonstrated a partial response (i.e. potential benefit) or stable disease (i.e. indeterminate benefit)

Patient ID	Drugs tested	n	%TGI	%TR	Overall response
LON-002	Paclitaxel*	3	110	19	PR/SD
	Capecitabine	3	68	N/A	PD
	GDC0032	3	73	N/A	PD
	Carboplatin/ Paclitaxel	3	94	N/A	PD
LON-008	Methotrexate	3	14	-152	PD
	Cetuximab*	3	107	13	SD
	Paclitaxel/ Cetuximab*	3	105	11	SD
	Gemcitabine	3	65	-81	PD
	Paclitaxel*	3	93	-17	SD
LON-009	Cisplatin	5	26	-207	PD
	Paclitaxel	5	-50	-436	PD
	5-fluorouracil	5	42	-173	PD
	Cetuximab	5	-1	-281	PD
LON-011	Gemcitabine	4	82	-36	PD
	Cisplatin	4	85	-25	PD
	Paclitaxel	4	-165	-432	PD
	Doxorubicin	4	-50	-132	PD
